# The fungus *Clonostachys epichloë* alters the influence of the *Epichloë* endophyte on seed germination and the biomass of *Puccinellia distans* grass

**DOI:** 10.3389/fmicb.2023.1146061

**Published:** 2023-06-26

**Authors:** Karolina Górzyńska, Paweł Olejniczak, Ewa Węgrzyn

**Affiliations:** ^1^Department of Systematic and Environmental Botany, Adam Mickiewicz University, Poznań, Poland; ^2^Institute of Nature Conservation, Polish Academy of Sciences, Kraków, Poland

**Keywords:** seed-borne fungi, vertical transmission, fungal microbiome, grass endophyte, mycoparasite

## Abstract

The fungal grass endophyte *Epichloë typhina* (Pers.) Tul. & C. Tul. (Ascomycota: Clavicipitaceae) grows intercellulary in aerial plant parts and reproduces asexually by invading host seeds. In this phase, it enhances seed production and germination, which accelerates its vertical spread. This relationship may be distorted by other seed-born fungi, whose spread is not so directly dependent on the success of the grass. Recently, the fungus *Clonostachys epichloë* Schroers has been observed on *Puccinellia distans* (Jacq.) Parl seeds originating from grass clumps infested with stromata, sexual structures of *Epichloë typhina* that are formed in spring on some host culms, preventing flower and seed development (‘choke disease’). *C. epichloë* shows mycoparasitic activity toward *Epichloë* stromata by reducing the production of ascospores, which are responsible for horizontal transmission of the fungus. The aim of this study was to investigate the effect of seed-borne *C. epichloë* on seed germination, as well as the size and weight of *P. distans* seedlings and to examine whether *C. epichloë* alters the influence of *Epichloë* in the early developmental stages of *P. distans*. The results showed that if *C. epichloë* acts on seeds together with *E. typhina* endophytes, the seeds were negatively affected due to the elimination of the positive effect of the latter in terms of both seed germination rate and seedling length. At the same time, *C. epichloë* increased the proportion of *E. typhina*-untreated germinated seeds. Additionally, only the joint action of the two fungi, *E. typhina* and *C. epichloë,* effectively stimulated seedling dry mass; the presence of *E. typhina* alone was not sufficient to noticeably affect seedling size. Based on the increasing commonality of *C. epichloë* on *Epichloë* stromata, as well as its potential to be used in biocontrol of ‘choke disease’, we should take a closer look at this fungus, not only in terms of its mycoparasitic ability, but also in terms of its cumulative impact on the whole *Epichloë-*grass system.

## 1. Introduction

Germination and seedling development are crucial stages that need to be successful for the growth of a new plant (Wolny et al., 2018). Because of the plant’s high vulnerability to injury, disease, and environmental stress during germination, this is considered to be the most critical phase in the plant life cycle. Seed-borne fungi are also factors that influence the process of germination (Shahzad et al., 2018).

Among grasses, fungi of the genus *Epichloë* are widespread and in addition to being seed-borne, they are also asymptomatic systemic grass endophytes (Tadych et al., 2014). These fungi grow between cells in aerial plant parts without showing any visible signs of infection. The fungus may penetrate generative reproductive plant organs and the ovule by gradually overgrowing in their tissues. After fertilization, the ovule develops into an infected seed (Gagic et al., 2018). This is one of two ways in which the *Epichloë* fungus spreads, i.e., vertical transfer (Schardl, 1996). In spring, some *Epichloë* species may form external stromata that form around the developing plant inflorescences, thereby suppressing their maturation and seed production. The mating system of *Epichloë* species has been shown to be bipolar heterothallic, whereby conidia produced on stromata are vectored by symbiotic *Botanophila* sp. flies between opposite mating types of the stromata (Bultman and Leuchtmann, 2008). Fungal mating takes place on stromata, upon which ascospores capable of infecting new grass plants are produced (Chung and Schardl, 1997). This type of spreading is known as horizontal transfer. In some *Epichloë*-grass associations, stromata can be fertilized without the fly contribution (Rao and Baumann, 2004; Górzyńska et al., 2010, 2011), and an ascosporic fertilization mechanism (Alderman and Rao, 2008) has been identified. There are 34 species of *Epichloë* that have been described so far (Leuchtmann et al., 2014) and each species exhibits one of three modes of reproduction: (1) exclusively *via* seeds, (2) exclusively *via* stromata development, or (3) a mixed mode where both flowering culms are choked by stromata and healthy flowering culms with seeds colonized by endophytic mycelium are present (Tadych et al., 2014).

*Epichloë* endophytes have been reported to increase the ability of the host grass to persist when exposed to biotic and abiotic stresses during its vegetative phase (Xia et al., 2018; Laihonen et al., 2022); however, some studies have shown that the endophyte-mediated effect on host plants is dependent on many factors, such as plant and fungal genotypes (Cheplick, 2008), plant nutrient content (Malinowski et al., 2000) and age of the grass host (Olejniczak and Lembicz, 2007). Moreover, some species of the genus *Epichloë* produce external sexual structures that hamper seed production (‘choke disease’) and cause economic losses in agricultural production and grass yields raised for seeds (e.g., Pfender and Alderman, 2006). For that reason, we cannot draw firm conclusions about the nature of *Epichloë* endophytes, and at present, *Epichloë*-grass interactions are thought to be variable and range from antagonistic to mutualistic (Saikkonen et al., 2004). Similarly, the role that *Epichloë* fungi play in host seed germination is ambiguous. For example, *Epichloë* endophytes were found to increase seed germination in *Lolium perenne* and *Festuca arundinacea* (Clay, 1987), *Bromus setifolius* (Novas et al., 2003) and *Achnatherum inebrians* (Chen et al., 2016). Conversely, some studies showed that *Epichloë* endophytes have no effect on the seed germination of *Festuca arizonica* (Faeth et al., 2004), and the endophytes can even decrease the germination rates of the *Epichloë*-infected seeds of *Lolium multiflorum* (Gundel et al., 2006) and *Puccinellia distans* (Czarnoleski et al., 2013). Other studies have reported that interactions between endophytes and their local habitats (Wäli et al., 2009) or origin of the host (Bao et al., 2019) can affect the germination of *Festuca rubra*, *F. ovina* or *Achnatherum inebrians* seeds. Seed germination may also be influenced by allelopathic interactions from other neighboring plants, an example of which is the reported allelopathic potential of *Pedicularis kansuensis* (Bao et al., 2015). The extracts of this weed inhibited both the seed germination and the seedling growth of *Stipa purpurea* and *Elymus tangutorum.* However, the extracts had a much weaker effect on the germination of *Epichloë*-infected seeds and they significantly inhibited the germination of seeds without endophytes. Identification of factors that alter the role of *Epichloë* fungi in the germination and seedling growth processes of the grasses they colonize is crucial for understanding variation in the frequency of endophyte-infection in grass populations.

The presence of other fungi of various ecological statuses, including both seed- and soil-borne, may additionally alter the influence of *Epichloë* fungi on the germination of the seeds of their host. To date, there has been little research focusing on the interaction of *Epichloë* with other fungi within (on/in) the host seed. One study found a significant difference between the number of seeds colonized by other fungi in the *Epichloë-*infected (Epi+) and *Epichloë*-free (Epi−) seed groups, implying that the presence of the *Epichloë* endophyte may influence the colonization of other fungi (Górzyńska et al., 2017). Other studies have examined the effect of seed-borne fungi such as *Alternaria alternata, Bipolaris sorokiniana* and *Fusarium avenaceum* in the presence of *Epichloë* endophytic fungi, including their effects on the germination of *Elymus sibiricus* grass seeds (Li et al., 2017). These seed-borne fungal species, known as parasites, were found to increase seed germination in the presence of *Epichloë* fungi. Fungi of the genus *Epichloë* also positively affect the diversity of the rhizosphere microbiome, including fungi and bacteria. Research by Roberts and Ferraro (2015) showed that rhizospheres of endophyte-infected plants (*Festuca arundinacea*) have higher species richness (Shannon diversity index = 4.03) than endophyte-free rhizospheres (Shannon diversity index = 3.08). Additionally, infection of grass with the fungus *Epichloë* increased the colonization of arbuscular mycorrhizal fungi (AMF) in the soil, e.g., *Bromus auleticus* plants infected with endophytes with the addition of *Epichloë tembladerae* extracts presented more arbuscules (Vignale et al., 2018). Vignale et al. (2018) also showed that endophyte-infected plants that were supplemented with *E. tembladerae* exudates presented higher values of shoot dry weight in the presence of AMF. Although the relationship between the quantity of AMF in the soil and the germination of seeds of grasses infected/not infected with *Epichloë* has not been investigated, their relationship seems likely, especially when considering that they had an impact on the previously mentioned characteristics of the plant.

**Figure 1:**
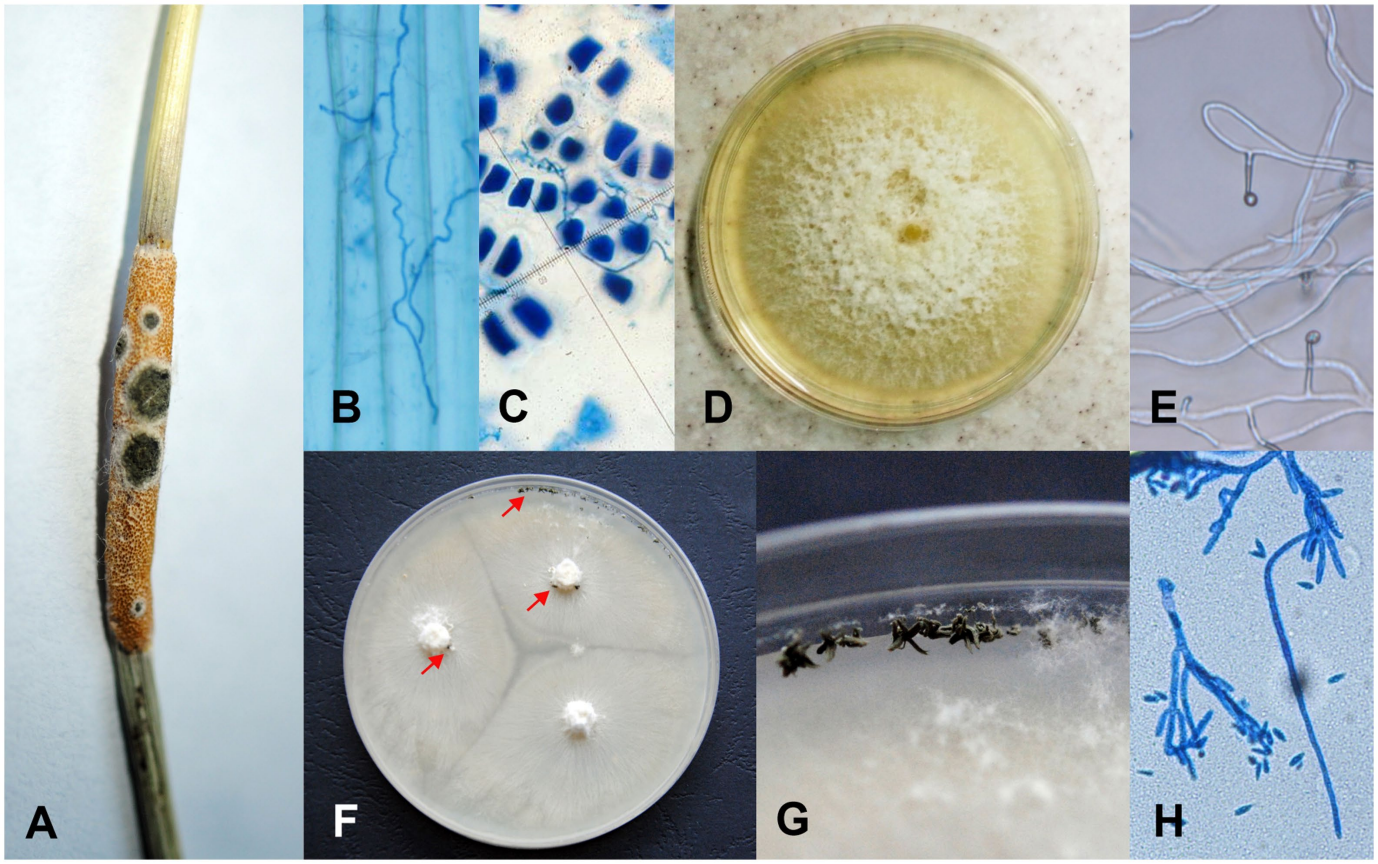
Life stages of *Clonostachys epichloë* and *Epichloë typhina* fungi. **(A)** *C. epichloë* (dark green spots) infecting the stromata of *E. typhina* on the grass *Puccinellia distans*. Endophytic hyphae of *E. typhina* **(B)** growing in a *P. distans* leaf and **(C)** penetrating the aleurone layer of a *P. distans* seed. **(D)** The colony of *E. typhina* from cultures grown on PDA at 25°C for 28 days. **(E)** *E. typhina* – conidiogenous cells with emerging conidia. **(F,G)** Colonies of *C. epichloë* from cultures grown on OA at 25°C for 28 days; arrows indicate conidial masses produced by sporodochia. **(H)** Divergently branched conidiophores of *C. epichloë*.

One of the fungi with which *Epichloë* has a close relationship with is *Clonostachys epichloë* Schroers. *C. epichloë* was identified on stromata – the sexual stage of *Epichloë typhina* (Figure 1A) – where it exhibited mycoparasitic activity by reducing the sexual reproduction and ascospore production of *E. typhina* (Górzyńska et al., 2018). Additionally, the entomopathogenic activity of *C. epichloë* against *Epichloë*-associated *Botanophila* flies was recently reported (Górzyńska, 2020). The fungus reduced the larval hatching success and increased the mortality of larvae present on the *Epichloë* stromata. This may lead to lower numbers of *Botanophila* adults in the next season, which may lead to a decrease in the sexual reproductive success of the fungus in these *Epichloë-Botanophila* systems in which the fungus is exclusively dependent on *Botanophila* flies.

Many species of the genus *Clonostachys* are also classified as plant endophytes (Schroers, 2001). Although no hyphae of *C. epichloë* have been found inside grasses or seeds (unpublished), it has been found in an epiphytic form on the leaves and seeds of the alkaligrass *P. distans*. This may indicate that it belongs to the group of seed-borne fungi that, unlike *Epichloë*, appear only on the surface of the seeds and not inside them. The influence of *C. epichloë* on the fungus *Epichloë* – including its presence in *Puccinellia* seeds and its impact on seed germination – has not been studied thus far.

In this study, we investigated the effect of seed-borne *C. epichloë* on seed germination, as well as the size and weight of *P. distans* seedlings. By using two seed groups, including those treated and not treated with the *Epichloë* endophyte, we were also able to examine whether *C. epichloë* alters the influence of *Epichloë* in the early developmental stages of *P. distans*.

## 2. Materials and methods

### 2.1. Study system

*Puccinellia distans* (Jacq.) Parl. is a perennial Euro-Siberian halophyte occurring on marine and inland salines (Hughes and Hallidays, 1980). Since the 1960s *P. distans* has colonized anthropogenic habitats of Central Europe (Jackowiak, 1996). The first report of an *Epichloë* fungus in a *P. distans* grass population is from 1992 from a single population in central Poland (Lembicz, 1996) and it was identified as *Epichloë typhina* (Pers.) Tul. & C. Tul. (Lembicz et al., 2011). The frequency of infected grass individuals was 7.5% in 1992 and in 1996 it was already 67.2%. *E*. *typhina* is the only species of the genus *Epichloë* reported so far in connection with *P. distans.* Fungal hyphae of *E*. *typhina* grow throughout the above-ground tissues of the grass (Figure 1B) and this genus represents a mixed mode of reproduction – both *via* ascospores produced on sexual stromata (Figure 1A) and *via* seeds colonized by endophytic mycelium (Figure 1C; Tadych et al., 2014).

The association between *E. typhina* and *P. distans* has already been investigated in several studies. The endophytic stage of *E. typhina* has a positive effect on the growth (Czarnoleski et al., 2013) and reproduction (Olejniczak and Lembicz, 2007) of *P. distans*, whereas the sexual stage leads to the partial or complete sterilization of the host (Lembicz et al., 2011). In addition, *E. typhina* on *P. distans* in sexual reproduction is not mutually dependent on the presence of *Botanophila* flies (Górzyńska et al., 2011). In 2018, the first report of the presence of *Clonostachys epichloë* fungus in *P. distans-E. typhina* system was published and its effect on the sexual reproduction of *E. typhina* has since been described (Górzyńska et al., 2018). Preliminary research showed that *C. epichloë* is also present on the surface of *P. distans* seeds originating from grass clumps infested with *Epichloë* stromata. For that reason, as well as the fact that *E. typhina* on *P. distans* also reproduces by seeds, we chose this grass-fungal system to investigate how *C. epichloë* alters the influence of *Epichloë* in the early developmental stages of *P. distans*. This system gives us a chance to elucidate the effect of *C. epichloë* on the grass-fungus system as a whole.

### 2.2. Fungus *Clonostachys epichloë* in *Puccinellia distans* grass populations

To assess the prevalence of *C. epichloë* in *P. distans* grass populations, material was collected in the spring and summer 2018 at three weeping alkaligrass sites in Poland – Janikowo (N 52°46.384′; E 18°08.032′), Pakosc2 (N 52°47.293′; E 18°06.721′) and Giebnia (N 52°46.544′; E18°06.190′). At each site, from 50 labeled grass individuals infected with *E. typhina* at the sexual stage, the following material was collected: *Epichloë* stromata (all present), 9 leaves and 3 inflorescences with mature seeds. Collections were placed directly in separate envelopes and transported to the laboratory.

The presence of the *C. epichloë* fungus on the stromata was determined with a binocular – this fungus forms a dark green sporodochia on them (Figure 1A). To determine the presence of the fungus on the surface of the seed and grass leaves, the samples were rinsed in autoclaved distilled water and then the water samples were placed on potato dextrose agar (PDA) medium (A&A Biotechnology). The presence of the *Clonostachys* fungus inside the seeds and grass leaves was determined using the following two methods: (1) by staining with aniline blue and microscopic analysis (Bacon and White, 1994) and (2) by placing them after surface sterilization (75% ethanol 5 min, sodium hypochlorite 10 min, 3 times rinsed in H_2_O) into the PDA medium. Plates with waters from the seed and leaf rinses and plates with seeds and leaf fragments were incubated under constant heat conditions (25°C) in the dark and continuously monitored for 28 days. Emerging mycelia were each transferred to a new plate, incubated at 25°C in the dark and monitored for the next 28 days. After, each strain was analyzed and identified based on morphological characters such as conidiophores and conidial sizes and shape comparisons with information provided by Schroers (2001) and Kirschner (2006) for *Clonostachys* and White (1993) for *Epichloë* (since the material was collected from grass individuals with a visible sexual stage of *Epichloë*, we expected its presence also in the endophytic form; Figures 1E,H). Additionally, these two fungal species are easy to distinguish in culture because *E. typhina* grows slower and forms colonies that are white on the upper surface and white to yellow on the reverse (Figure 1D), whereas *C. epichloë* forms mycelia with green conidial masses (Figures 1F,G).

In summary, for each of the 150 grass individuals from all three sites, the following attributes were evaluated for the presence of *Clonostachys*: all collected stromata; the inside of 3 leaves and 3 seeds using the microscopic method; the inside of 3 leaves and 3 seeds using culture in plates; and the surface of 3 leaves and 3 seeds. A positive result (presence of *C. epichloë*) for a given individual on/in a given organ was considered if at least one sample was positive.

### 2.3. The influence of the two species of fungi on the germination and size of seedlings – an experiment

#### 2.3.1. Field harvesting

At the Pakość2 site, seeds with the *Epichloë* endophyte (Epi+) and *Epichloë* stromata with visible symptoms of *C. epichloë* infection were collected from 30 *P. distans* grass individuals. Seeds that were not infected with *Epichloë* (Epi−) were collected from 30 *P. distans* individuals located nearby where no *Epichloë* stomata were recorded. The seeds were mixed within their categories, and 50 seeds from each group were checked for the presence of *Epichloë* to confirm the correct isolation of the two seed collections, namely Epi+ and Epi−. The presence of *Epichloë* was verified using the two methods mentioned in section 2.2.

#### 2.3.2. Preparation of *Epichloë typhina* supernatant

Thirty seeds were randomly selected from the Epi + group, which included samples previously collected at the Pakość2 station. Seeds were sterilized (75% ethanol for 5 min, sodium hypochlorite for 10 min, washed 3 times in H_2_O) and then placed on PDA plates in an incubator (25°C in the dark). The emerging mycelium was transferred to new plates and incubated for 14 days under the same conditions. After, the mycelium disc was transferred to potato dextrose broth (PDB) medium with a cork borer and incubated again for 28 days (25°C in the dark). Then, the broth was filtered and centrifuged, and the filtrate (4 × 15 mL) was diluted 50%.

#### 2.3.3. Preparation of *Clonostachys epichloë* spore suspension

Isolates of *C. epichloë* were collected from *E. typhina* stromata by touching a drop of sterile water to the sporulating surface and spreading the conidia that adhered to the drop over the surface of PDA plates with a glass rod. Mycelia were incubated at 25°C. After 2 weeks, they were transferred to oatmeal agar OA medium and incubated for 1.5 months to induce conidia production. Conidia were collected in sterile distilled water and transferred to sterile tubes. Suspensions were adjusted to a concentration of 106 spores ml^−1^ using a Thoma hemocytometer.

#### 2.3.4. Experiment

Sterilized Epi− grass seeds (method as above) were divided into 4 groups (A, B, C, D) of 150 seeds each. In each group, the seeds were then divided into three subgroups of 50 seeds each and placed in separate falcon tubes. To each group A and B test tube, 15 mL of *Epichloë* supernatant was added, and to each group C and D tube, 15 mL of autoclaved distilled water was added (Table 1). All tubes were sealed with parafilm and placed in an incubator (25°C) for 12 h in the dark. After, 40 seeds from each tube were placed in sterile petri dishes lined with sterile filter paper. Five milliliters of the suspension containing *Clonostachys* spores was added to the plates with seeds from groups A and C, and 5 mL of autoclaved distilled water was added to the plates with seeds from groups B and D. In this way, the categories listed in the last column of Table 1 were defined. Then, the dishes were placed in a phytotron, and the seeds were watered every other day (5 ml of distilled, autoclaved H_2_O).

**Table 1:** Seed categories used in the experiment.

Group	Number of seeds	(Epi/H_2_O)	(Clo/H_2_O)	Category
A	3 × 40 (120)	Epi	Clo+	Epi+ Clo+
B	3 × 40 (120)	Epi	H_2_O	Epi+ Clo−
C	3 × 40 (120)	H_2_O	Clo+	Epi− Clo+
D	3 × 40 (120)	H_2_O	H_2_O	Epi− Clo−

The germinated seeds were counted daily at the same time each day. The germination potential (7 days after sowing) and the percentage of seeds germinated (after 14 days) were measured. On the 14th day after sowing, all the seedlings were harvested, and the above-ground and below-ground lengths were measured using an electronic caliper. Then, seedlings were placed individually in small envelopes, dried and weighed with an accuracy of 0.0001 g (A&D, HM-120).

### 2.4. Analysis

The effects of the presence of *Epichloë* and *Clonostachys* fungi on *P. distans* seed germination and seedling growth were analyzed by a two-way ANOVA. Two dichotomous presence/absence variables (Epi+, Epi− and Clo+, Clo−) were used as grouping variables, and their interaction was also included in the model. The number of germinated seeds on a dish recorded on the 7th and 14th days after sowing, the mean length of below- and above-ground parts per dish, and the mean seedling dry mass per dish at day 14 were all dependent variables in the analyses. Prior to the analyses, the homogeneity of variance in each dependent variable was checked using Bartlett’s test. When the effect of the interaction between grouping variables was significant, a one-way ANOVA was conducted separately for the Clo+ and Clo− subsets. All statistical analyses were performed using Dell Statistica, ver 13.1.

## 3. Results

### 3.1. The fungus *Clonostachys epichloë* in *Puccinellia distans* grass populations

The occurrence of *C. epichloë* on *P. distans* was high at all sites, and it was present on *Epichloë* stromata and on *P. distans* seeds and leaves (Table 2). There were no reports of *C. epichloë* inside the plants, either in seeds or in leaves. The presence of *C. epichloë* on seeds was associated with its presence on *Epichloë* stromata, i.e., in all individuals with recorded *C. epichloë* on seeds, it was also present on *Epichloë* stromata.

**Table 2:** Presence of *C. epichloë* fungus on/in leaves and seeds of *P. distans* and on *E. typhina* stromata infecting *Puccinellia* shoots at three field sites.

	Janikowo	Pakosc2	Giebnia	Overall
*Epichloë* stromata	45 (90%)	47 (94%)	40 (80%)	(88%)
*P. distans* leaves – inside	0 (0%)	0 (0%)	0 (0%)	(0%)
*P. distans* leaves – surface	49 (98%)	47 (94%)	50 (100%)	(97%)
*P. distans* seeds – inside	0 (0%)	0 (0%)	0 (0%)	(0%)
*P. distans* seeds – surface	42 (84%)	46 (92%)	35 (70%)	(82%)

The numbers of infected samples and their frequency within each group are given (*n* = 50 in each group).

### 3.2. The influence of the two species of fungi on the germination and size of seedlings

Variances in all dependent variables did not deviate from homogeneity across experimental groups (Bartlett’s tests, df = 3; germination rate at day 7, *ꭓ*^2^ = 0.857, *p* < 0.836; germination rate at day 14, *ꭓ*^2^ = 1.824, *p* < 0.610; aboveground length *ꭓ*^2^ = 3.968, *p* < 0.265; belowground length, *ꭓ*^2^ = 5.096, *p* < 0.165; seedling dry mass *ꭓ*^2^ = 2.810, *p* < 0.422). The presence of both *Epichloë* and *Clonostachys* fungi affected the development of *P. distans* seeds, but the effect was not clear, as shown by a significant Epi × Clo interaction for the number of germinated seeds on day 7 (Table 3A) and day 14 (Table 3B), and the seedling mass (Table 3E).

**Table 3:** Results of two-way ANOVA for (A) germination rate at day 7, (B) germination rate at day 14, (C) above-ground seedling length, (D) below-ground seedling length and (E) seedling dry mass.

Dependent variable	Two-way ANOVA	One-way ANOVA	
Source of variation		Clo−	Clo+
**(A) Germination rate 7D**			
Intercept	407.76***	240.07***	175.62***
Presence of *Epichloë*	0.12	6.90*	3.46
Presence of *Clonostachys*	0.12		
*Epichloë* × *Clonostachys*	9.82**		
**(B) Germination rate 14D**			
Intercept	604.41***	252.49***	371.69***
Presence of *Epichloë*	0.33	4.71*	1.05
Presence of *Clonostachys*	0.74		
*Epichloë* × *Clonostachys*	5.38*		
**(C) Above-ground length**			
Intercept	1450.46***		
Presence of *Epichloë*	8.44**		
Presence of *Clonostachys*	4.60*		
*Epichloë* × *Clonostachys*	0.01		
**(D) Below-ground length**			
Intercept	903.42***		
Presence of *Epichloë*	5.13*		
Presence of *Clonostachys*	3.35		
*Epichloë* × *Clonostachys*	0.04		
**(E) Seedling mass**			
Intercept	1938.66***	693.92***	1371.69***
Presence of *Epichloë*	10.60**	0.61	16.93***
Presence of *Clonostachys*	14.47***		
*Epichloë* × *Clonostachys*	4.24*		

When the two-way interaction was significant, a one-way ANOVA was performed separately for seeds exposed to *Clonostachys* (Clo+) and the control (Clo−). *F* values and their significances are given, ***p < 0.001, **p < 0.01, *p < 0.05.

**Figure 2:**
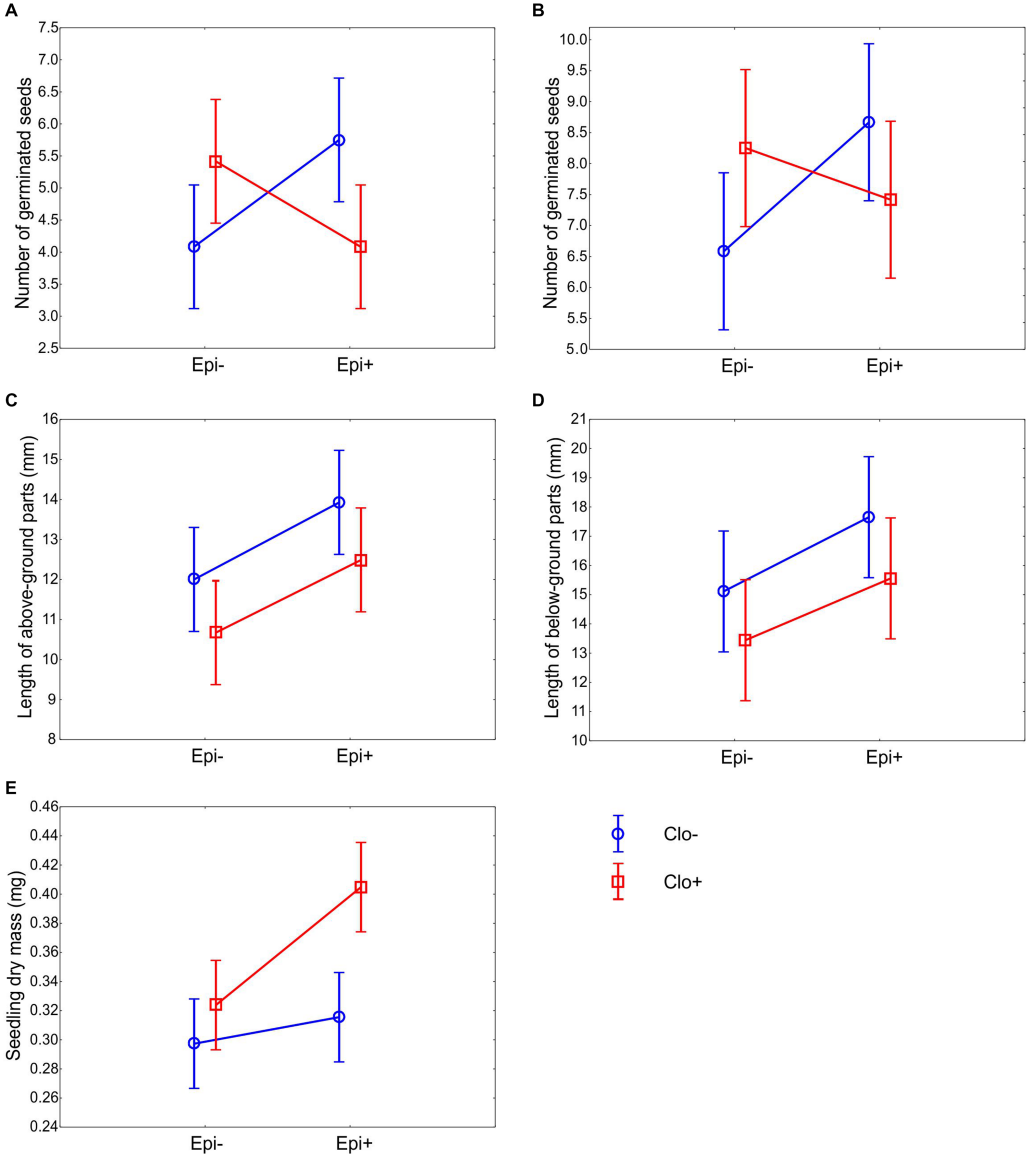
Effects of the presence of *E. typhina* and *C. epichloë* on seed germination and seedlings of *P. distans*. Means ±95% confidence intervals are shown for number of seeds that germinated at day 7 **(A)** number of seeds that germinated at day 14 **(B)** length of above-ground parts **(C)** length of below-ground parts **(D)** and seedling dry mass **(E)**. Twelve petri dishes with ten seeds each were used in every experimental group. Seedling measurements were taken on the 14th day after sowing.

The positive effect of one of the fungal species on the germination rate of *P. distans* seeds was counterbalanced by the presence of the other species. The probability of seed germination was higher in *Epichloë*-infected seeds only in the absence of *Clonostachys,* and conversely, *Epichloë*-free seeds germinated better in the absence of *Clonostachys*. This effect was observed both on day 7 (Figure 2A) and on day 14 (Figure 2B). The positive effect of *Epichloë* on Clo− seed germination was significant (Tables 3A,B), whereas its negative effect on Clo+ seed germination was not.

The effect of the fungi on seeds that had already germinated was different. The presence of *Epichloë* stimulated the growth of above-ground seedling parts regardless of the presence of *Clonostachys*, whereas the presence of *Clonostachys* reduced the length of above-ground parts in both the Epi− and Epi+ groups (Figure 2C; Table 3C: significant effects of *Epichloë* and *Clonostachys*, nonsignificant *Epichloë* × *Clonostachys* interaction). An analogous pattern was identified for the length of the below-ground parts of the seedlings. The below-ground parts in Epi+ plants grew significantly longer than those in Epi− plants (Figure 2D; Table 3D); however, the negative effect of the presence of *Clonostachys* on this trait (Figure 2D) was nonsignificant (Table 3D).

Seedling dry mass was affected by the presence of both *Epichloë* and *Clonostachys* fungi (Table 3E). *P. distans* seedlings grew the most when both fungal species were present. Moreover, only in the Epi+ Clo+ group did the 95% confidence limits not overlap with those of the other groups (Figure 2E). Thus, the presence of only one of the fungi alone was not sufficient to exert noticeable effects on seedling size, and only their joint action could effectively stimulate seedling growth.

## 4. Discussion

The results of our study demonstrate that (1) *C. epichloë* eliminated the positive effect of *E. typhina* on seed germination but increased the proportion of germinated seeds when alone; (2) *E. typhina* alone did not have a noticeable effect on seedling size, and only the joint action of the two fungi, *E. typhina* and *C. epichloë,* could effectively stimulate seedling growth. Previous studies on *C. epichloë* have focused on its anti-fungal activity against *Epichloë* species (Steinebrunner et al., 2008a; Górzyńska et al., 2018), as well as its entomopathogenic activity against *Epichloë-*associated *Botanophila* flies (Górzyńska, 2020). Although *C. epichloë* was found in association with plants in the past, it was actually associated with dead rachises of ferns (*Pteridium aquilinum*) and grasses (e.g., *Sasa* sp.) (Schroers, 2001). Thus, this is the first study investigating the effect *C. epichloë* on a living plant and its early developmental stages.

The frequency of *C. epichloë* on *Epichloë* stromata has seemingly increased. The known distribution of the fungus *C. epichloë* was only the Neotropics, the United States and Japan for a long time, and it was noted there not only on *Epichloë* stromata but also on stromata of *Balansia* sp. and on dead rachises of ferns and grasses (Schroers, 2001). The first report of *C. epichloë* in Europe was provided by Kirschner (2006), who described it on specimens of *E. typhina* collected on *Dactylis glomerata* grass in Germany. The presence of *C. epichloë* on *Epichloë* stromata was also confirmed in Switzerland (Steinebrunner et al., 2008a). In these reports, there is no information on the scale of the phenomenon but we have accurate data on this subject from Poland. In 2010, during field work in populations of *P. distans* grass infected with *E. typhina*, we reported a previously unrecorded *C. epichloë* on fungal stromata (Górzyńska et al., 2018). At that time, *C. epichloë* was present on 8.5% of all collected *Epichloë* stomata, whereas the frequency in the same sites in the present study (2018) was 88%. The factors affecting the spread of the fungus are unknown. Flies associated with *Epichloë* stromata appear on them earlier than the fungus, hence their role as a vector of *C. epichloë* has been excluded (Górzyńska, 2020). Undoubtedly, atmospheric factors such as temperature and humidity have an influence in the case of fungi, but they have not been measured. However, we do know for sure that *Epichloë* fungi secrete volatiles, which reduce the spore germination of fungal hyperparasites (Steinebrunner et al., 2008a), and patterns of odor profiles appeared to be largely dependent on particular *Epichloë*-host associations (Steinebrunner et al., 2008b), which may further complicate the spread of *C. epichloë*. An explanation of the increasing frequency of infection requires further research. This is important due to previous reports and research on the potential use of mycoparasites present on *Epichloë* stromata as biocontrol agents limiting its spread (Alderman et al., 2010; Górzyńska et al., 2018). Such agents must not negatively affect the other elements of the interaction, i.e., the fungal host – grass.

Our results indicate that *C. epichloë* positively influences the germination of *P. distans* seeds by increasing the proportion of germinated seeds, but only when acting individually. Furthermore, when considering this positive effect of *C. epichloë*, its relationship with *Epichloë* stromata should also be taken into account. It is very likely that, at least in our case, the *C. epichloë* on *Puccinellia* seeds comes from the *Epichloë* stromata, where it lives as a mycoparasite (Górzyńska et al., 2018). This assumption is supported by our results. In all of the samples with *C. epichloë* identified on seeds, it was also identified on *Epichloë* stromata. At the same time, the presence of *Epichloë* stromata on grass shoots indicates the presence of this fungus in seeds, as *E. typhina* utilizes a mixed mode of endophyte reproduction, where both flowering culms choked by stromata and healthy flowering culms with seeds colonized by endophytic mycelium are present (Tadych et al., 2014). Thus, assuming that both fungi coexist in nature within one grass specimen, we are interested in the effect of *Clonostachys* in the presence of *Epichloë* rather than the effect of *Clonostachys* alone. If it acts on seeds together with *E. typhina,* it exerts a negative effect by eliminating the positive effect of the latter. Although not 100% of grass seeds are usually infected due to imperfect transmission (Afkhami and Rudgers, 2008), in *P. distans,* the infection frequency of seeds produced by tillers that were known to host *E. typhina* was always very high (unpublished data). In such situations, the number of Epi− Clo+ seeds is too low to compensate for the losses resulting from the negative impact of *Clonostachys* in Epi+ Clo+ seeds. Thus, the final effect of the presence of *C. epichloë* in the *P. distans* population will depend on the percentage of seeds infected with *Epichloë,* which is influenced not only by the number of infected grass individuals but also by the effectiveness of vertical transmission. Endophyte seed-transmission efficiency is influenced by host genetics (Gagic et al., 2018) and by plant defense responses, which reduce endophyte colonization of host reproductive tissues (Zhang et al., 2022). According to Afkhami and Rudgers (2008), *Epichloë* endophytes can be lost at all possible stages: within adult plants, from adult tillers to seeds, and from seeds to seedlings, and the type and degree of loss differed not only among species and endophyte genera but among host populations as well, which makes estimating the number of infected seeds very difficult.

It is also worth considering the further fate of such seeds. Seeds treated with *Clonostachys* but deprived of *Epichloë* (Epi− Clo+) will grow into seedlings that are also free of *Epichloë*. Such plants will not suffer from the parasitic stage of *Epichloë –* stromata – in the later stages of development, but according to our results, *Clonostachys* will not also benefit them, as two fungal species are needed to increase seedling growth. Seeds with two fungi (Epi+ Clo+) will grow into larger seedlings, but they will also suffer from the parasitic stage of *Epichloë,* and stromata will choke their inflorescences. Further studies are needed to determine whether *Clonostachys* may survive on the developing plant and then “attack” *Epichloë* stromata as a mycoparasite. This would not have a direct effect on the infested grass at this stage, but more broadly, it is possible that *Clonostachys*, by reducing the number of infectious ascospores on stromata, will reduce the horizontal transmission of *Epichloë* and the overall impact of its presence will be positive.

The effect of *Epichloë* on the germination of grass seeds in the presence of other seed-borne fungi has already been studied by Li et al. (2017). They showed that *Epichloë* supernatant increased the parameters of seed germination and the size and weight of *Elymus sibiricus* grass seedlings in the presence of *Alternaria alternata, Bipolaris sorokiniana, Fusarium avenaceum* and *Fusarium* sp. Regardless of the supernatant’s origin – three *Epichloë* species were used – and the seed-borne species fungus always increased the percentage of germinated seeds and the size of the seedlings. These results are in part different from ours as in our study, seeds exposed to simultaneous treatment with *Epichloë* and *Clonostachys* germinated like control seeds, without any fungus. Conversely, similar to Li et al. (2017), seedlings from such seeds were the heaviest, although the lengths of the under-ground and above-ground parts were similar to the control. The reason for this difference can be explained by the ecology of the seed-borne fungi used in these two experiments – *A. alternata, B. sorokiniana, F. avenaceum* and *Fusarium* sp. are known pathogens that negatively affect seed germination and plant growth (Kamil et al., 2020). The effect of *C. epichloë* on the plant has not been studied thus far and, as it turns out, it is similar to the effect of *Epichloë* – both fungi acting alone increase seed germination and increase seedling size. Therefore, it is not a pathogen for the plant, but it is a mycoparasite for the *Epichloë* inhabiting the plant. The impact of *C. epichloë* on the *Epichloë* host plant is important because, like many other mycoparasites, *C. epichloë* has the potential to be used as a biocontrol agent. Mycoparasitism is widespread in nature (Jeffries and Young, 1994; Jeffries, 1995) and has been intensely studied as a potential biocontrol measure in fungal diseases. For instance, the mycoparasite *Sphaerellopsis filum* decreases the winter survival of *Puccinia graminis* in perennial ryegrass (Gordon and Pfender, 2012). *Clonostachys rosea* has potential as a biocontrol agent for the control of alfalfa blossom blight caused by *B. cinerea* (Li et al., 2004), while *Sphaerodes retispora* may play a similar role against watermelon wilt caused by *Fusarium oxysporum* (Harveson et al., 2002). *C. epichloë* reduces the ascospore production of *E. typhina* (Górzyńska et al., 2018) and thus limits the effectiveness of its horizontal transmission, which may have a positive impact on limiting the spread of the economically important ‘choke disease’ in grass populations. As biocontrol agents used at large scales must not cause any harm to the plants that undergo treatment, it is necessary to investigate their impacts on each element of this tritrophic interaction. Such research can give surprising results, as in the case of the interaction between *Pseudozyma flocculosa* in the context of its biocontrol activity against *Blumeria graminis* – a parasite of *Hordeum vulgare* (Laur et al., 2018) in which *P. flocculosa* can actually transiently parasitize the plant.

In summary, the increasing frequency of *C. epichloë* on *Epichloë* stromata supports the idea that we should take a closer look at this fungus, not only in terms of its mycoparasitic ability, but also in terms of its cumulative impact on the *Epichloë*-grass system.

## Data availability statement

The raw data supporting the conclusions of this article will be made available by the authors, without undue reservation.

## Author contributions

KG formulated the idea, performed the field work and conceived, designed and performed the experiments. KG and PO analyzed the data. KG wrote the first draft of the manuscript. KG, PO, and EW wrote sections of the manuscript. All authors contributed to the article and approved the submitted version.

## Funding

This work was supported by the National Science Centre grant number UMO 2014/13/D/NZ8/02420. The cost of publication was financed from the funds of the “Excellence Initiative - Research University” (ID-UB) UAM program (application no. 085/08/POB1/0016, competition no. 085).

## Conflict of interest

The authors declare that the research was conducted in the absence of any commercial or financial relationships that could be construed as a potential conflict of interest.

## Publisher’s note

All claims expressed in this article are solely those of the authors and do not necessarily represent those of their affiliated organizations, or those of the publisher, the editors and the reviewers. Any product that may be evaluated in this article, or claim that may be made by its manufacturer, is not guaranteed or endorsed by the publisher.
